# mHealth Interventions for Lifestyle and Risk Factor Modification in Coronary Heart Disease: Randomized Controlled Trial

**DOI:** 10.2196/29928

**Published:** 2021-09-24

**Authors:** Jang-Whan Bae, Seoung-Il Woo, Joongyub Lee, Sang-Don Park, Sung Woo Kwon, Seong Huan Choi, Gwang-Seok Yoon, Mi-Sook Kim, Seung-Sik Hwang, Won Kyung Lee

**Affiliations:** 1 Division of Cardiology, Department of Internal Medicine Chungbuk National University Hospital Chungbuk National University College of Medicine Cheongju Republic of Korea; 2 Department of Cardiology, School of Medicine Inha University Hospital Inha University Incheon Republic of Korea; 3 Department of Preventive Medicine Seoul National University College of Medicine Seoul Republic of Korea; 4 Medical Research Collaborating Center Seoul National University Hospital Seoul Republic of Korea; 5 Department of Public Health Sciences Graduate School of Public Health Seoul National University Seoul Republic of Korea; 6 Department of Prevention and Management, School of Medicine Inha University Hospital Inha University Incheon Republic of Korea

**Keywords:** coronary heart disease, prevention, lifestyle modification, mobile health, text message, mHealth

## Abstract

**Background:**

Self-management of lifestyle and cardiovascular disease risk factors is challenging in older patients with coronary heart disease (CHD). SMS text messaging could be a potential support tool for self-management and the most affordable and accessible method through a mobile phone. High-quality evidence had been lacking, and previous studies evaluated the effects of SMS text messaging on the subjective measures of short-term outcomes. Recently, a large-sized randomized controlled trial in Australia reported promising findings on the objective measures upon 6-month follow-up. However, an examination of the effectiveness of such interventions in an Asian population with unique demographic characteristics would be worthwhile.

**Objective:**

This study examined the effectiveness of a 1-way SMS text messaging program to modify the lifestyle and cardiovascular disease risk factors of patients who underwent the first percutaneous coronary intervention (PCI).

**Methods:**

A parallel, single-blinded, 1:1 random allocation clinical trial was conducted with 879 patients treated through PCI. They were recruited during hospital admission from April 2017 to May 2020 at 2 university hospitals in the Republic of Korea. In addition to standard care, the intervention group received access to a supporting website and 4 SMS text messages per week for 6 months regarding a healthy diet, physical activity, smoking cessation, and cardiovascular health. Random allocation upon study enrollment and SMS text messaging after hospital discharge were performed automatically using a computer program. The coprimary outcomes were low-density-lipoprotein cholesterol (LDL-C), systolic blood pressure (SBP), and BMI. The secondary outcomes were change in lifestyle and adherence to the recommended health behaviors.

**Results:**

Of the eligible population, 440 and 439 patients who underwent PCI were assigned to the intervention and control groups, respectively. The 1-way SMS text messaging program significantly enhanced physical activity (*P*=.02), healthy diet (*P*<.01), and medication adherence (*P*<.04) among patients with CHD. Hence, more people were likely to control their cardiovascular disease risk factors per the recommendations. The intervention group was more likely to control all 5 risk factors by 62% (relative risk 1.62, 95% CI 1.05-2.50) per the recommendations. On the other hand, physiological measures of the primary outcomes, including LDL-C levels, SBP, and BMI, were not significant. Most participants found the SMS text messaging program useful and helpful in motivating lifestyle changes.

**Conclusions:**

Lifestyle-focused SMS text messages were effective in the self-management of a healthy diet, exercise, and medication adherence, but their influence on the physiological measures was not significant. One-way SMS text messages can be used as an affordable adjuvant method for lifestyle modification to help prevent the recurrence of cardiovascular disease.

**Trial Registration:**

Clinical Research Information Service (CRiS) KCT0005087; https://cris.nih.go.kr/cris/search/detailSearch.do/19282

## Introduction

A healthy lifestyle, risk factor modification, and medication adherence are vital for preventing mortality and recurrent events in individuals with coronary heart disease (CHD). Previous studies have revealed the preventive effect of lifestyle modifications including smoking cessation, exercise, healthy diet, and exercise on mortality among patients with CHD. A Cochrane systematic review demonstrated that exercise-based cardiac rehabilitation reduced the all-cause mortality by 26% [1]. Another systematic review also reported that smoking cessation could reduce the risk of death and myocardial infarction in patients with CHD by 30% [2]. In the 2017 factsheet, the World Health Organization reported that approximately 75% of recurrent vascular events might be prevented when the patients were adherent to medications, such as aspirin, β-blockers, angiotensin-converting enzyme inhibitors (ACEis), and statin, and practices such as smoking cessation [3].

On the other hand, self-management of the cardiovascular disease risk factors is often challenging. A previous study reported that approximately one-third of patients with acute coronary syndrome persisted in smoking or were not adherent to the recommendations for diet or exercise [4]. Therefore, the development of tools to enhance self-management and evaluation of their effectiveness is crucial. Texting using a mobile phone has been suggested as a potential tool because it is affordable and accessible to older people with CHD [5]. Early research suggested the possible benefit of texting interventions on lifestyle modification [6]. On the other hand, a Cochrane review pointed out that evidence was not strong for such interventions because of the small number of participants and the risk of bias [7]. Moreover, previous studies were limited to the short-term consequence of texting interventions and the subjective measures on the outcomes [7-9]. Recently, Chow et al [10] reported encouraging findings in a large-scale RCT that a text message program could change the objective measure of cardiovascular disease risk factors and the self-reported lifestyle upon 6-month follow-up. On the other hand, it is unclear if such interventions would be effective in an Asian population with a different culture and lifestyle.

Therefore, this study examined whether an SMS text messaging program could enhance self-management of lifestyle and risk factor modification with objective and subjective measures in a randomized controlled trial.

## Methods

### Study Design

This study is a parallel, single-blind, randomized controlled trial that enrolled 879 patients with a 1:1 allocation. The care provider and outcome evaluator were blinded to the assignment. Demographic information was obtained using a questionnaire at baseline. The following objective measures of the coprimary outcomes were obtained at baseline and 6 months after enrollment: low-density-lipoprotein cholesterol (LDL-C) levels, systolic blood pressure (SBP), and BMI. Subjective measures of the secondary outcomes included a self-report of physical activity (PA), diet, and medication adherence in the questionnaire.

The institutional review boards of the Inha University Hospital (IRB number 2017-03-008-001) and the Chungbuk National University Hospital (IRB number 2017-05-016) approved the study protocol, and informed consent was provided by patients who participated in this study. The protocol of this trial was registered retrospectively at the Clinical Research Information Service of the Republic of Korea (registration number KCT0005087).

### Participants

Participants were eligible if they were diagnosed with CHD and underwent percutaneous coronary intervention (PCI) for the first time; those younger than 18 years were excluded. Initially, acute myocardial infarction was targeted as the inclusion criterion, but the criteria were extended to CHD, including angina pectoris, treated through PCI after the participating hospitals were confirmed at 1 month of recruitment. A cardiologist diagnosed the participants with CHD after coronary angiography and PCI during hospitalization. The patients were excluded if they had no mobile phone or difficulty reading SMS text messages. The demographic characteristics were evaluated during hospitalization. The income level was obtained through subjective assessment of household income. The participants selected 1 of the following five levels: low, middle-low, middle, middle-high, and high household income.

Participants were recruited after face-to-face assessment during hospital admission at 2 tertiary and university teaching hospitals in Chungcheongbuk-Do and Incheon, Republic of Korea. The study areas, Chungcheongbuk-Do and Incheon, had a population of 1,590,372 and 2,922,121 individuals, respectively, in 2020. Both hospitals had regional cardiocerebrovascular centers (RCCVCs) established by the Ministry of Health and Welfare to prevent and treat cardiovascular disease [11].

### Intervention

Access to a supporting website and SMS text messages regarding lifestyle modification were provided for 6 months to the intervention group. The contents of the SMS text messages were based on the Tobacco, Exercise, and Diet Messages (TEXTME) trial and the Australian Heart Foundation Healthy Living Guidelines [10,12]. The cardiologists, nurses, clinical nutritionists, and preventive medicine experts reviewed the text messages in the TEXTME trial and modified them considering the Asian diet and culture. The SMS text messages related to smoking cessation, diet, physical activity, and general cardiovascular health, including medication adherence. The number of messages was 25 and 27 for the category of smoking cessation and physical activity, respectively. The diet category consisted of 27 and 99 messages for vegetarian and nonvegetarian individuals, respectively. General cardiovascular health categories A and B included 24 messages for all participants and nonsmokers. Category A included 24 messages for heart health and medication adherence, and category B included 24 mixed messages regarding diet, physical activity, general heart health, and information on passive smoking. Category B was designed to prevent nonsmokers from receiving duplicate messages from category A. The message-sending program delivered semipersonalized text messages considering the smoking status and diet pattern of the participants—vegetarian or not—with their names.

Participants in the intervention group received 4 messages per week for 24 weeks in addition to standard care. The message-management program selected a message randomly from each of the following four categories for a week: smoking cessation, physical activity, diet, and general cardiovascular health A for smokers; and physical activity, diet, and general cardiovascular health A and B for nonsmokers. The messages were sent on 4 of 5 randomly selected weekdays and at randomly selected times (9 AM, 12 PM, 3 PM, and 5 PM) of the day. Two text messenger systems were used: the default text messenger of the mobile phone and a commercial messenger (Kakaotalk), which is the most popular messaging app in the Republic of Korea. Text message delivery was ensured by programming the system to send the message to the commercial messenger first and then to the default messenger if the text message could not be delivered. The text messaging program re-sent the message via the default text messenger. Every participant in the intervention group received 96 messages in the 6-month prevention program. The algorithm for message management was developed in accordance with the prespecified rule on selecting the categories and text messages and the frequency and timing of texting. The participants were not meant to reply to the messages because they were designed as a 1-way SMS text messaging program. The participants were told not to respond to the messages and were informed that the messages were managed using a computer program. On the other hand, the study personnel explained to all the participants that they could request to stop the SMS text messaging program through the caller’s phone number when they wanted to withdraw.

The supporting websites were the homepages of Chungcheongbuk-Do and Incheon RCCVC [13,14]. They provided information on cardiovascular disease, including a healthy lifestyle and disease management. Nine common action plans were selected as a preventive lifestyle of cardiovascular disease by 14 RCCVCs: quit smoking, avoid heavy drinking, reduce salt intake, exercise regularly, maintain an ideal body weight, reduce stress in daily life, take a regular health examination, adhere to medical treatment, and call an emergency response system if there are symptoms of cardiovascular disease. These were reflected in the contents of the leaflets, booklets, infographics, and video clips on the supporting websites. The study participants in the intervention group received the website link on their mobile phones. Although the website was introduced and recommended to the intervention group, a visit to the website was not required or checked.

### Outcomes

The coprimary outcome of the study was the LDL-C level, SBP, and BMI at 6 months, relative to baseline levels. Fasting lipid levels, systolic and diastolic blood pressure with the heart rate, and BMI were measured in accordance with international standardized procedures at baseline and at 6 months. Blood pressure and heart rate were measured using electronic devices (Exhomax plus HBP-1000, HuBDIC Healthcare Co). Three resting measurements in sitting position with digital recordings were taken, each at least 5 minutes apart, with the mean of the last 2 readings used for analyses. The body weight and height were determined using an automatic standardized scale (GBF-500, TransTek) and electronic height rod (BSM330, InBody) with an accuracy of 100 g and 1 mm, respectively. BMI was calculated by dividing the weight in kilograms by the square of the height in meters. Regarding obesity, a BMI of 25 kg/m^2^ was used as a cut-off level in accordance with the Asia Pacific guidelines of obesity of the World Health Organization [15]. The lipid levels (LDL-C, high-density-lipoprotein cholesterol, and triglycerides) of the participants were measured from the fasting blood samples and are reported as mg/dL.

The secondary outcomes were healthy lifestyle at the 6-month follow-up: smoking cessation, PA, fruit and vegetable intake up to ≥2 times/day, and medication adherence. At the 6-month visit to the cardiology clinic, smoking status, PA, diet, and medication adherence were acquired using a questionnaire. PA was assessed using a shortened Korean version of the International Physical Activity Questionnaire (IPAQ), a commonly used tool to assess PA. The total hours per week for walking, moderate PA, and vigorous PA were computed using metabolic equivalent (MET) values [16]. As a secondary outcome, PA was reported as the median METs-min/week and the proportion of inactive PA. PA was categorized into active and inactive on the basis of the recommendations of the total level: ≥600 and <600 METs-min/week.

Past and current smoking statuses were assessed through a self-report, including the duration and quantity of smoking and the duration of smoking cessation. The proportion of current smokers among the participants in the intervention and control groups upon 6-month follow-up was compared to that at baseline.

The frequency of fruit and vegetable intake over the last 7 days was assessed in the questionnaire: “How many days or times in the last 7 days did you consume fruit or vegetables?” The participants could select 1 of seven answers: 0 times, 1-2 times/week, 3-4 times/week, 5-6 times/week, 1 time/day, 2 times/day, ≥3 times/day. They were reclassified into ≥2 times/day and <2 times/day in the analysis. Medication adherence was evaluated using the Modified Morisky Scale (MMS) and the number of days the medication was taken per month [17]. Medication adherence was defined as good if the participants took their medication as instructed on 25 or more days in the last month, corresponding to more than 80%. A 6-item MMS was used to assess medication-taking behavior: 2 questions were added to the validated 4-item Morisky Scale to explain the persistence of therapy [18]. The score ranged from 0 to 3 for every 2 domains (knowledge and motivation); the total score ranged from 0 to 6. As a secondary outcome, medication adherence was reported using the proportion of good medication adherence, and the median MMS score was also presented.

Moreover, the secondary outcomes included the proportion achieving the guideline levels of 5 modifiable risk factors: LDL-C levels of <70 mg/dL, blood pressure of <140/90 mmHg, 30 minutes of moderate exercise up to ≥5 days/week, smoking cessation, and BMI of <25 kg/m^2^ [10,19]. The effects of the SMS text messaging program on the risk factor modification were evaluated by dividing the participants into those who met all 5 guideline levels and those who did not meet ≤4. Similarly, participants were also classified with the cut-off levels of any 4 and 3 guidelines achieved: ≥4 guideline level and <4 guideline level; ≥3 guideline level and <3 guideline level.

### Program Evaluation

In this study, the intervention was a 1-way SMS text messaging program, including a supporting website, and the study participants were asked to not reply to the messages. The program was evaluated by administering a questionnaire to the intervention participants to assess the perceived utility, acceptability, and influence of the intervention on behavioral changes. The questionnaire also included questions on the reading, saving, and sharing of text messages, but it was not validated (Multimedia Appendix 1). These were given to participants at the outpatient clinic after the final outcome assessment, and 349 participants conducted a self-report. The participants reported the level agreement to the statements related to the usefulness and acceptability of the text messages using a 5-point Likert scale: 1=strongly disagree, 2=disagree, 3=neither agree nor disagree, 4=agree, and 5=strongly agree. For analysis, “agree” and “strongly agree” were reclassified as “agree,” while all the other categories were classified as “disagree.”

### Sample Size

A sample size of 880 individuals was calculated considering a 15% loss to follow-up, 90% power (2-tailed; significance level=5%) to detect a difference in the 3 primary outcomes between the 2 groups: 10 mg/dL in LDL-C levels, 5 mmHg in SBP, and 1.2 kg/m^2^ in BMI [10]. For the coprimary outcomes, no significance level adjustment was made to the sample size calculations to account for multiple comparisons.

### Randomization

After a pilot study, a computerized randomization program was developed for a random 1:1 allocation whose sequence was generated in a block size of 8. A web-based interface was developed for computerized randomization and an SMS text message–sending program. When the study personnel entered the participant information in the secure web interface, the participants were assigned randomly to the intervention or control group. If they were assigned to the intervention and discharged from the hospital, the computer program automatically sent an SMS text message for 6 months after hospital discharge. The study personnel could not ask the participants about their allocation. The study participants were asked to not reveal their allocation at the 6-month follow-up visit to maintain blinding of the study personnel. Except for SMS text messages, both groups received guideline-directed standard care for CHD: treatment including medication, regular follow-up at the outpatient clinic, and education on cardiovascular health and risk factors provided by nurses specialized in patient education at RCCVCs.

### Statistical Analysis

Statistical analysis was conducted by an independent biostatistician and an epidemiologist not involved in the study. All evaluations of the intervention were performed on the principle of an intention to treat. Subgroup analyses were specified in the statistical analysis plan by age, sex, smoking status at baseline, recruiting hospital, LDL-C tertile at baseline, and CHD category: acute myocardial infarction and angina pectoris. No interim analysis was planned or conducted.

Continuous variables at baseline are presented as mean (SD) values if they were distributed normally, while the median (IQR) values are used to describe the nonnormally distributed variables. Categorical variables are presented as n (%) values. The baseline characteristics between the intervention and the control groups were compared using an independent samples *t* test and chi-square test. The primary analysis was an analysis of covariance (ANCOVA) and robust Poisson regression to estimate the relative risk with the baseline values of the analyzed parameters, where continuous and binary outcomes were evaluated, respectively [20]. A randomization-based nonparametric ANCOVA was used when the continuous outcomes were not normally distributed.

Regarding the coprimary outcomes, the results were not statistically significant. An adjustment to account for multiple comparisons, such as a Bonferroni correction, was not needed to reduce the overall false-positive rate.

The analyses were conducted using SAS Enterprise Guide (version 7.4, SAS Institute). All statistical tests were 2-tailed, and *P*<.05 was considered significant.

### Data availability

Data are available from the authors upon reasonable request.

## Results

### Results Overview

**Figure 1:**
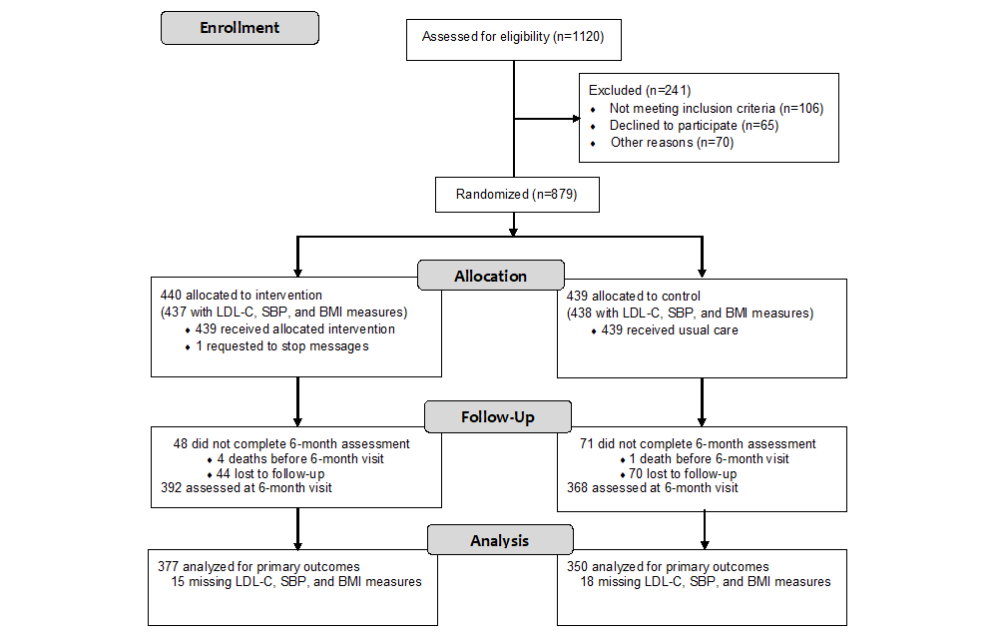
Schematic representation of the randomized controlled trial. LDL-C: low-density-lipoprotein cholesterol, SBP: systolic blood pressure.

Of the 1120 patients screened for eligibility, 879 who were admitted with CHD and underwent PCI from April 2017 to May 2020 were enrolled in this study (Figure 1). In total, 241 patients were excluded: 28 patients who did not have a mobile phone, 78 patients who had difficulty reading SMS text messages, 65 patients who declined to participate in the study, and 70 patients who declined participation for other reasons, including cases of in-hospital mortality. Of the 879 eligible patients, 440 and 439 patients were assigned randomly to the intervention and control groups, respectively. In total, 48 patients were lost to follow-up, including 4 patients who died before the 6-month follow-up in the intervention group, while 71 patients in the control group were lost to follow-up, including 1 death. Recruitment was concluded when the study sample size was achieved, and the follow-up period was from October 2017 to November 2020.

### Baseline Characteristics of the Study Participants

Table 1 lists the baseline characteristics of the study participants. The mean age of the enrolled participants was 60.4 (SD 10.5) years, and 732 (83.3%) participants were male. The mean LDL-C level, SBP, and BMI were 110.3 mg/dL, 125.3 mmHg, and 24.9 kg/m^2^, respectively. The baseline characteristics were similar in the intervention and control groups except for the ACEis/angiotensin receptor blockers (ARBs); the control group was more likely to take ACEis or ARBs before hospital admission.

**Table 1:** Baseline characteristics of the study participants (N=879).

Characteristics			Intervention (n=440)		Control (n=439)		Total
**Demographics**							
	Age (years), mean (SD)	60.1 (10.6)		60.7 (10.4)		60.4 (10.5)	
	Males, n (%)	368 (83.6)		364 (82.9)		732 (83.3)	
**Participating institution, n (%)**							
	Inha university hospital	323 (73.4)		323 (73.6)		646 (73.5)	
	Chungbuk university hospital	117 (26.6)		116 (26.4)		233 (26.5)	
**Education level, n (%)**							
	Elementary school or less	51 (11.7)		48 (11.0)		99 (11.3)	
	Middle school	55 (12.6)		55 (12.6)		110 (12.6)	
	High school	205 (46.9)		207 (47.4)		412 (47.1)	
	University	102 (23.3)		99 (22.7)		201 (23.0)	
	Graduate school or higher	24 (5.5)		28 (6.4)		52 (6.0)	
**Income, n (%)**							
	Low	57 (13.0)		49 (11.2)		106 (12.1)	
	Middle-low	58 (13.3)		67 (15.3)		125 (14.3)	
	Middle	251 (57.4)		246 (56.3)		497 (56.9)	
	Middle-high	56 (12.8)		49 (11.2)		105 (12.0)	
	High	15 (3.4)		26 (6.0)		41 (4.7)	
**Clinical data**							
	Acute myocardial infarction, n (%)	219 (50.1)		212 (49.3)		431 (49.7)	
	BMI (kg/m^2^), mean (SD)	25.0 (3.4)		24.9 (3.1)		24.9 (3.2)	
	Waist circumference (cm), median (IQR)	90 (85-96)		90 (85-95)		90 (85-96)	
	Hip circumference (cm), mean (SD)	94.2 (10.3)		94.0 (11.2)		94.1 (10.7)	
	Total cholesterol (mg/dL), median (IQR)	174 (145-209)		171 (145-201)		172 (145-205)	
	Low-density-lipoprotein cholesterol (mg/dL), mean (SD)	110.8 (41.2)		109.8 (38.1)		110.3 (39.7)	
	High-density-lipoprotein cholesterol (mg/dL), median (IQR)	43 (37-51)		43 (36-50)		43 (37-51)	
	Triglycerides (mg/dL), median (IQR)	124 (89-186)		125 (90-181)		125 (90-185)	
	Systolic blood pressure (mmHg), mean (SD)	124.4 (18.9)		126.1 (19.8)		125.3 (19.4)	
	Diastolic blood pressure (mmHg), mean (SD)	74.0 (12.4)		74.7 (13.0)		74.3 (12.7)	
	Heart rate (beats/min), mean (SD)	75.3 (12.3)		76.5 (12.8)		75.9 (12.6)	
Risk factor level: low-density-lipoprotein cholesterol level ≥ 70 mg/dL, n (%)			358 (81.9)		372 (84.9)		730 (83.4)
**Blood pressure, n (%)**							
	Systolic > 140 mmHg	90 (20.6)		75 (17.2)		165 (18.9)	
	Diastolic > 90 mmHg	43 (9.8)		33 (7.6)		76 (8.7)	
BMI ≥ 25 kg/m^2^, n (%)			200 (45.8)		205 (46.8)		405 (46.3)
Physical activity (metabolic equivalents-min/week), median (IQR)			0 (0-720)		0 (0-924)		0 (0-777)
Inactive (<600 metabolic equivalents-min/week), n (%)			312 (71.4)		293 (67.1)		605 (69.2)
**Smoking status, n (%)**							
	Current	189 (43.3)		184 (42.1)		373 (42.7)	
	Former	145 (33.2)		146 (33.4)		291 (33.3)	
Diabetes, n (%)			127 (29.0)		129 (29.5)		256 (29.2)
Hypertension, n (%)			212 (48.4)		212 (48.4)		424 (48.4)
**Achieving guideline levels, n (%)**							
	Low-density-lipoprotein cholesterol level < 70 mg/dL	79 (18.1)		66 (15.1)		145 (16.6)	
	Blood pressure < 140/90 mmHg	330 (75.5)		306 (69.9)		636 (72.7)	
	Exercising regularly^a^	125 (28.6)		144 (33.0)		269 (30.8)	
	Nonsmokers	248 (56.8)		253 (57.9)		501 (57.3)	
	BMI < 25 kg/m^2^	237 (54.23)		233 (53.20)		470 (53.71)	
	All 5 key guideline levels	8 (1.8)		8 (1.8)		16 (1.8)	
	Achieving 4 of 5 key guideline levels	55 (12.6)		59 (13.5)		114 (13.0)	
**Medications before admission, n (%)**							
	Angiotensin-converting enzyme inhibitor/angiotensin receptor blocker	130 (29.7)		158 (36.1)		288 (32.9)	
	Aspirin	103 (23.5)		118 (26.9)		221 (25.2)	
	β-Blocker	48 (11.0)		44 (10.1)		92 (10.5)	
	Statin	117 (26.7)		122 (27.9)		239 (27.3)	
	All 4 medications	10 (2.3)		16 (3.7)		26 (3.0)	

^a^Regular exercise involves more than 30 minutes of moderate exercise performed ≥5 days/week.

### Study Outcomes

The SMS text messaging program yielded modest improvement in the primary outcomes, whereas it improved the PA, diet, and medication adherence significantly (Table 2). Although the LDL-C level tended to decrease in the intervention group, the differences between the 2 groups were not significant for any of the 3 outcomes: LDL-C levels, SBP, and BMI. On the other hand, PA was significantly higher in the intervention group than in the control group, by 220 (95% CI 36-404) min/week. Participants in the intervention group were more likely to eat fruit or vegetables frequently (≥2 times a day) and adhere to their medications (≥80%) compared to those in the control group.

**Table 2:** Primary and secondary outcomes upon 6-month follow-up.

Parameters			Values					Mean difference (95% CI)			*P* value
			Intervention (n=377)		Control (n=350)						
**Primary outcomes**											
	Low-density-lipoprotein cholesterol level (mg/dL), mean (95% CI)	73.9 (70.8 to 77.0)		77.4 (74.2 to 80.6)		–3.6 (–8.0 to 0.9)			.12		
	Systolic blood pressure (mmHg), mean (95 CI)	126.6 (125.0 to 128.2)		128.4 (126.7 to 130.0)		–1.8 (–4.1 to 0.5)			.13		
	BMI (kg/m^2^), mean (95% CI)	25.0 (24.9 to 25.2)		25.1 (25.0 to 25.3)		–0.09 (–0.3 to 0.1)			.41		
**Secondary outcomes^a^**											
	Current smokers, n (%)	79 (20.6)^b^		81 (23.0)^b^		0.98 (0.76 to 1.3)^c^			.86		
	Physical activity (metabolic equivalents-min/week), median (IQR)	693 (0 to 1386)		384 (0 to 1162)		220 (36 to 404)			.02		
	Inactive (<600 metabolic equivalents-min/week), n (%)	173 (45.2)^b^		208 (59.1)^b^		0.75 (0.66, 0.86)^c^			<.001		
	Fruit/vegetable intake ≥ 2 times/day^d^, n (%)	89 (23.2)^b^		52 (14.8)^b^		1.5 (1.1 to 2.1)^c^			.006		
	Medication adherence > 80%, n (%)	376 (98.2)^b^		324 (92.1)^b^		1.1 (1.0 to 1.1)^c^			<.001		
	Modified Morisky Scale score^e^, median (IQR)	5 (5 to 5)		5 (5 to 5)		0.07 (–0.03 to 0.16)			.19		

^a^Regarding the secondary outcome, 383 and 352 participants in the intervention and control groups, respectively, responded to the follow-up survey.

^b^Unadjusted proportion of lifestyle in the control and intervention groups upon 6-month follow-up.

^c^Adjusted relative risk between the control and intervention groups.

^d^Denotes the frequency of fruit and vegetables consumed in the prior 7 days up to ≥2 times/day.

^e^The number of participants who responded to the Modified Morisky Scale upon 6-month follow-up were 380 and 352 in the intervention and control groups, respectively.

### Guideline Levels Achieved

The participants in the intervention group were more likely to achieve a healthy lifestyle by following the recommended guidelines (Table 3). The participants were categorized as those who met all 5 guideline levels and those who did not meet ≤4. The intervention group was more likely to control all 5 risk factors than the control group (relative risk [RR] 1.62, 95% CI 1.05-2.50). If the outcomes were set as meeting ≥4 guidelines, the participants in the intervention group were more likely to achieve the guideline level on ≥4 risk factors than those in the control group (RR 1.27, 95% CI 1.03-1.56). On the other hand, there was no significant difference in each risk factor between the intervention and control groups, except PA.

**Table 3:** Achieving guideline levels of risk factors upon 6-month follow-up.

Parameters		Intervention (n=378), n (%)	Control (n=350), n (%)	Relative risk (95% CI)	*P* value
Low-density-lipoprotein cholesterol level < 70 mg/dL		192 (50.8)	160 (45.7)	1.10 (0.95-1.28)	.19
Blood pressure < 140/90 mmHg		297 (78.6)	264/350 (75.4)	1.04 (0.96-1.12)	.38
Exercising regularly^a^		210^b^ (54.8)	144^b^ (40.9)	1.37 (1.17-1.60)	<.001
Nonsmoker		304^b^ (79.4)	271^b^ (77.0)	1.02 (0.95-1.08)	.64
BMI < 25 kg/m^2^		196 (51.9)	169 (48.3)	1.02 (0.91-1.16)	.71
**Key guideline levels**					
	Achieving all 5	48 (12.7)	27 (7.7)	1.62 (1.05-2.50)	.03
	Achieving ≥ 4	135 (35.7)	97 (27.8)	1.27 (1.03-1.56)	.02
	Achieving ≥ 3	278 (73.5)	229 (65.6)	1.11 (1.01-1.22)	.03

^a^Regular exercise involves more than 30 minutes of moderate exercise up to ≥5 days/week.

^b^Regarding physical activity and nonsmoking, 383 and 352 participants in the intervention and control groups, respectively, responded to the follow-up survey.

### Subgroup Analysis

**Figure 2:**
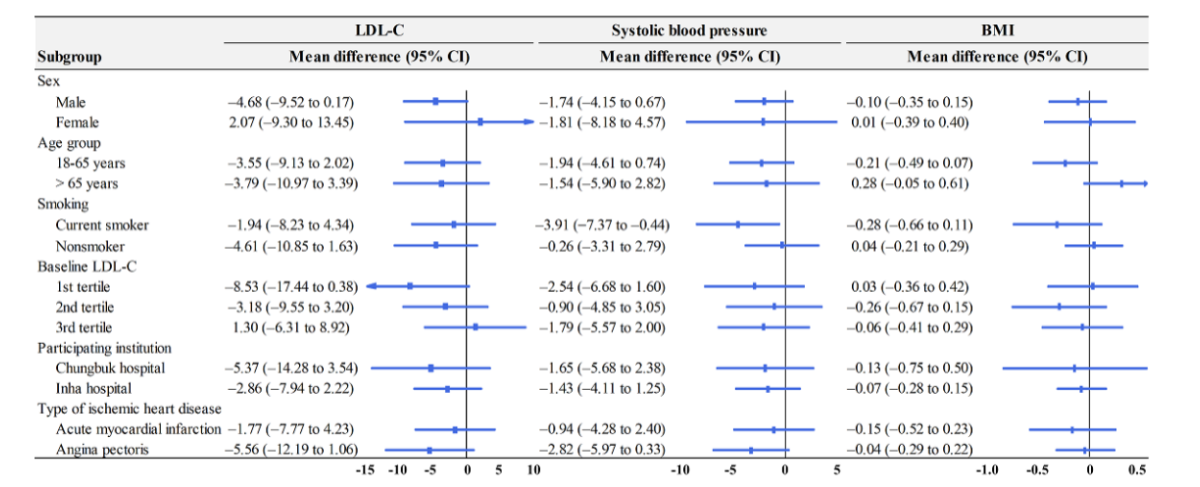
Subgroup analysis of the primary outcomes. LDL-C: low-density-lipoprotein cholesterol.

**Figure 3:**
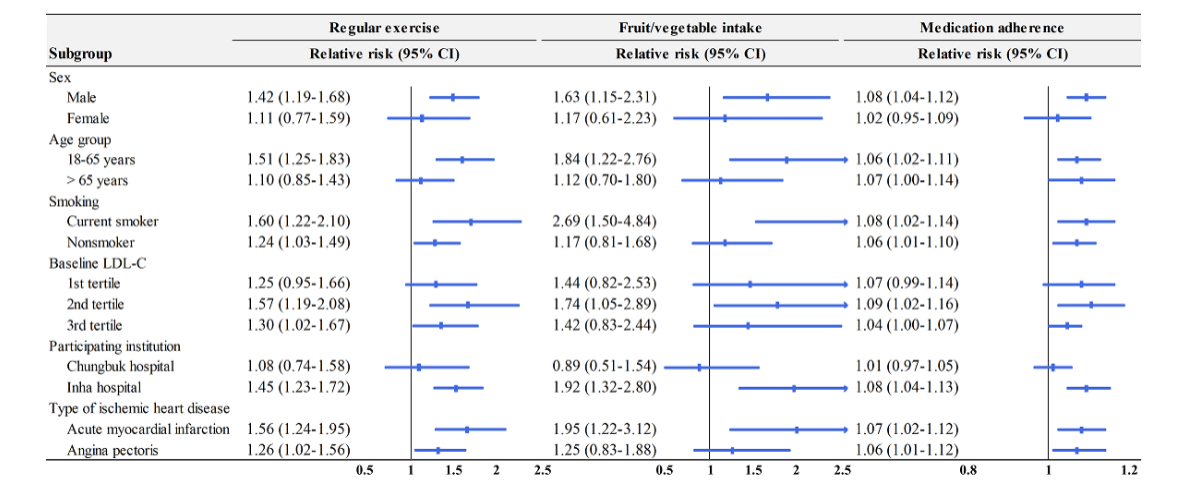
Subgroup analysis of the secondary outcomes: physical activity, fruit/vegetable intake, and medication adherence. LDL-C: low-density-lipoprotein cholesterol.

Figures 2 and 3 show the results of subgroup analysis on the primary and secondary outcomes. Regarding the objective measures of the primary outcomes, the difference between the intervention and control groups was not significant (Figure 2). On the other hand, with 3 secondary outcomes showing a significant difference between the intervention and control groups, the SMS text messages were more likely to be effective among males, young adults, current smokers, or patients with acute myocardial infarction (Figure 3).

### Program Evaluation

Among 440 participants in the intervention group, 349 completed the questionnaire on the utility and acceptability of the SMS text messaging program (Table 4). Most of the participants responded that the SMS text messages of the program were helpful (82.0%), easy to understand (94.6%), and a good motivation for changing their lifestyle (78.2%). Overall, they reported satisfaction with the frequency, time, and duration of the program. During the 6-month period, only 1 participant opted out of the SMS text messaging program. The cost of the SMS text messaging program was US $2.1 per person for regular SMS text messaging and US $0.5 per person using the commercial messenger app. The numbers of SMS text messages delivered through the default text messenger and the commercial text messenger were 20,658 (49%) and 21,502 (51%), respectively, from among 42,160 messages sent to the intervention group.

**Table 4:** Utility and perceived acceptability of the SMS text messaging intervention program by the participants (n=349).

Characteristics		Participants, n (%)
**Usefulness and understanding**		
	Found messages useful	286 (82.0)
	Messages were easy to understand	330 (94.6)
**Influence on motivation and behavior change** ** **		
	Messages motivated change	273 (78.2)
	Diet was more healthy owing to the messages	219 (62.8)
	Exercise increased owing to the messages	213 (61.0)
	Messages reminded to take medication	184 (52.7)
**Message saving and sharing** ** **		
	Read at least 80% of messages	318 (91.1)
	Saved messages	161 (46.1)
	Shared messages with family, friends, or clinicians	126 (36.1)
**Appropriate message characteristics** ** **		
	Number of messages per week	291 (83.4)
	Program length (6 months)	297 (85.1)
	Time of the day when messages were received	303 (86.8)

## Discussion

### Principal Findings

The 1-way SMS text messaging program and a supporting website enhanced physical activity and encouraged a healthy diet and medication adherence among patients who underwent PCI. Therefore, more people were likely to follow the lifestyle and risk factor modification as recommended. On the other hand, the intervention could not induce a significant decrease in each objective measure of risk factors: LDL-C levels, SBP, and BMI. Most participants found the SMS text messaging program a helpful motivation to change their lifestyle.

### Comparison With Prior Work

In this study, the SMS text messages contributed to lifestyle modification, but its impact on the physiological measures of risk factors may not be as much as those on lifestyle modification. These findings were inconsistent with those of the TEXTME study [10]—the clinical trial benchmarked by this study. The TEXTME study [10] reported significant improvement in all 3 objective measures. Regarding the objective measures, the mean differences between the intervention and control groups in this study were smaller than those in the TEXTME trial (LDL-C levels: 3.6 vs 5 mg/dL, SBP: 1.8 vs 7.6 mmHg, and BMI: 0.1 vs 1.3 kg/m^2^). This may be owing to different profiles of the lifestyle and risk factors of the study participants between the 2 trials; the participants in this study were older Asian people with a lower BMI and SBP but a more sedentary life than those in the TEXTME trial [10]. Moreover, at our study sites, nurses provided education and counseling about cardiovascular disease to all the patients during hospital admission, regardless of the allocation. This may reduce the gap between the intervention and control groups. For example, the decreases in LDL-C levels were more significant in both groups in this study than in the TEXTME study [10] (73.9 and 77.4 mg/dL in the control and the intervention groups, respectively, from a baseline of 110 mg/dL in this study, and 79 and 84 mg/dL, respectively, from a baseline of 101 mg/dL in the TEXTME study). Similarly, the control group of this study was more likely to quit smoking than that of the TEXTME study. The proportion of smokers decreased from 42% to 23% in the control group in this study and from 54% to 44% in the TEXTME study. This may contribute to the difference in the effect size between this study and the TEXTME trial.

This study shows that the SMS text messaging program was effective in improving the PA, diet, and medication adherence in patients with CHD. Regarding lifestyle modification, these findings were supported by those of previous trials [6]. Pfaeffli et al [17] reported that in an RCT, SMS text messages and a supporting website effectively promoted healthy behavior of PA, diet, alcohol, and smoking. Khonsari et al [21] showed that a short, automated message enhanced medication adherence in patients with acute coronary syndrome. On the other hand, the differences in LDL-C levels, SBP, and BMI between the intervention and control groups were not significant. Similar findings to those of our study were found, but there are only a few reports on the objective measures to draw a conclusion. Maddison et al [22] reported that SMS text and video messages could increase PA in leisure and walking and self-efficacy but did not enhance the objective measure of the exercise capacity—peak oxygen uptake—upon 24-week follow-up in the patients with CHD. Pfaeffli et al [17] also failed to demonstrate statistical significance regarding the BMI, waist to hip ratio, blood pressure, and LDL-C levels. Similarly, SMS text messaging helped remind the patients to take their medication without skipping but was ineffective in helping control SBP and LDL-C upon 6-month follow-up [23]. Therefore, further research will be needed to determine the effects of SMS text messaging programs on the physiological measures and clinical outcomes, such as recurrent cardiovascular events and mortality [21].

A method is needed to enhance the self-management of lifestyle and risk factors in patients with CHD. In this study, only a small proportion of patients who underwent PCI achieved the guideline level of all 5 risk factors: 12.7% for the intervention group and 7.7% for the control group. Although the SMS text messages improved PA and diet, many participants who underwent PCI still did not follow the guidelines. As much as 45.2% of the participants were considered physically inactive; only 23.3% of the participants ate fruit/vegetables up to ≥2 times per day in the intervention group. The corresponding proportion was 37.4% for physically inactive participants in the TEXTME trial [10]. Moreover, the proportion of patients who achieved all 5 guideline levels was worse in the TEXTME trial than in this study: 4.7% for the intervention group and 1.8% for the control group. This suggests that risk factor modification still has a long way to go, and various programs will be needed to improve lifestyle among patients with CHD.

SMS text messaging was chosen in this study because it was the most affordable and accessible among the elderly. It required lower cost and effort with and without an automated computer program than alternative methods. On the other hand, this study showed that its effect was not significant on the objective measures. Therefore, it is important to enhance the program by using interactive text messaging, personalized messaging, other smartphone apps, and wearable devices [24]. For example, previous review studies suggested that smartphone apps including goal-setting, self-monitoring of diet and activity, and feedback through SMS text messages could help lower calories, lower fat levels, increase PA, and reduce more weight in the general and individuals with obesity [8,25].

### Limitations

This study had some limitations. First, those without literacy or a mobile phone were excluded, which may be an entry bias. Although SMS text messaging is the most affordable and accessible method using mobile Health (mHealth), there could still be a barrier to individuals without literacy or a mobile phone [26]. Second, this study was conducted using a single-blind design, even though a computer program was developed for random allocation. The study personnel who collected the data upon 6-month follow-up were blinded to the allocation. Therefore, the self-reported measures of PA, diet, and medication adherence may be biased because the participants could be subject to the desirability and expectation of the research. Although the evaluator was blinded to the allocation, caution should be taken when interpreting positive subjective outcomes and negative objective outcomes. Similarly, program evaluation may have been subject to information bias because the utility and perceived acceptability were subjective and could be influenced by expectations, even though it was evaluated as a self-report. Therefore, the utility and acceptability of the program may also have been overestimated. Furthermore, the questionnaire on lifestyle was not comprehensive. For example, although the frequency of fruit or vegetable intake was queried, the portion of fruit or vegetable intake was not evaluated.

### Conclusions

This SMS text messaging program resulted in an improvement in self-reported lifestyle modifications such as PA, fruit and vegetable intake, and medication adherence among patients who required strict self-management after PCI. In contrast, its impact on physiological measures (LDL-C levels, SBP, and BMI) was not significant. One-way SMS text messaging may be used as an affordable adjuvant method for lifestyle modification to prevent the recurrence of cardiovascular disease. Because many patients still did not achieve guideline levels, future research will need to evaluate other interventions using mHealth tools (interactive messages, personalized messages, and wearable devices to facilitate the self-management of patient behaviors) with the objective measures of risk factor management.

## Appendix

Multimedia Appendix 1 Program evaluation.

Multimedia Appendix 2 CONSORT-eHEALTH checklist (V 1.6.1).

## Abbreviations

ACEi: angiotensin-converting enzyme inhibitor
ANCOVA: analysis of covariance
ARB: angiotensin receptor blocker
CHD: coronary heart disease
IPAQ: International Physical Activity Questionnaire
LDL-C: low-density-lipoprotein cholesterol
MET: metabolic equivalent
MMS: Modified Morisky Scale
PCI: percutaneous coronary intervention
RCCVC: regional cardiocerebrovascular center
RR: relative risk
SBP: systolic blood pressure
TEXTME: the Tobacco, Exercise, and Diet Messages
