# Supplementary material for: mHealth Interventions for Lifestyle and Risk Factor Modification in Coronary Heart Disease: Randomized Controlled Trial
Source: JMIR Mhealth Uhealth. 2021 Sep 24;9(9):e29928. doi: 10.2196/29928 (PMC8501404; doi:10.2196/29928)
Supplement: Multimedia Appendix 1 [file mhealth_v9i9e29928_app1.docx]

**Program Evaluation**

|  | Strongly agree | Agree | Neutral | Disagree | Strongly disagree |
| --- | --- | --- | --- | --- | --- |
| 1. Found the messages useful |  |  |  |  |  |
| 1. The messages were easy to understand |  |  |  |  |  |
| 1. The messages motivated me to change |  |  |  |  |  |
| 1. My diet is healthier due to the messages |  |  |  |  |  |
| 1. My Exercise increased due to the messages |  |  |  |  |  |
| 1. The messages reminded me to take medicines |  |  |  |  |  |
| 1. The number of messages per week was appropriate |  |  |  |  |  |
| 1. The program length was appropriate (6 months) |  |  |  |  |  |
| 1. The time of day the messages were received was appropriate |  |  |  |  |  |

1. I read ( )% of the messages. ① 0~20, ②21~40, ③41~60, ④61~80, ⑤81~100
2. I saved the messages. ① Yes, ② No
3. I shared the messages with family, friends, and/or clinicians ①Yes, ② No
4. The message language was
5. Too casual, ② Casual, ③Appropriate, ④Formal, ⑤Too Formal
